# The Evolutionary History of Renal Surgery and of Temporal Bone Surgery

**Published:** 1924-04

**Authors:** J. Lacy Firth

**Affiliations:** Surgeon to the Bristol General Hospital


*" Scire est nescire, nisi id me*
*Scire alius sciret."*

APRIL, 1924.

### THE EVOLUTIONARY HISTORY OF RENAL SURGERY AND OF TEMPORAL BONE SURGERY.

The Presidential Address, delivered on October 10th, 1923, at the opening of the Fifty-first Session of the Bristol Medico-Chirurgical Society.

BY

J. LACY FIRTH, M.D., M.S. Lond., F.R.C.S.,

*Surgeon to the Bristol General Hospital.*

### PART II.

MY next subject is the rise and development of operations on the mastoid process of the temporal bone. The importance of such operations for the relief of inflammation is very great, for they save many lives, much suffering, and much hearing power. It seems strange, therefore, that until about 1873 this branch of surgery remained very imperfectly developed.

In all surgical history two epochs have to be borne in mind, separated by a world of difference in the opportunity they offered their workers of making progress. I mean, of course, the epoch before and the epoch since the discovery of anæsthetics and antiseptics. We may compare these two periods to those before and after the dawn of Christianity in ethical history, and the middle years of the nineteenth century will for ever be memorable in human annals for those two great and beneficent discoveries.

It was in 1846 that Morton the dentist and Warren the surgeon, in America, first used ether as an anæsthetic. Within a year Simpson introduced chloroform. Liston performed an amputation through the thigh under ether at University College Hospital in 1847, and within eighteen months thousands of operations had been done under anæsthetics. Lister published his first work on antiseptics in 1867, but it was not until about 1880 that the antiseptic system of wound treatment became generally adopted. The dates 1846 and 1880 are those to be remembered when considering the history of mastoid or any other surgery.

In 1919 Sir Charles A. Ballance published a magnificent book on the *Surgery of the Temporal Bone*, and I have found the historical ground I wish to survey so well covered in that work that I have drawn largely from the excerpts he has taken from original papers by the pioneers of mastoid surgery. In addition, I am indebted to F. Whiting's book on *The Modern Mastoid Operation*, published in 1905, and to Politzer's *Geschichte der Ohrenheilkunde*, vol. i., published in 1907, and vol. ii. in 1913.

Until the middle of the nineteenth century aural surgery was in a very backward state in this country. It was largely in the hands of quacks.

As an illustration of this Sir Charles Ballance quotes the following story of Sir Robert Peel (b. 1788, d. 1850) and a notorious ear-quack named Curtis. The latter was summoned to attend Mr. Peel, the Secretary of State for the Home Department, who was suffering from a temporary deafness. One of Curtis's modes of practice, which he adopted in almost every case, was to clear out the affected organ by means of injecting warm water through an immense syringe. This instrument, which was not unlike one of Read's garden syringes, he carried down with him to the residence of his illustrious patient. On his arrival he found Mr. Peel in the drawing-room with the Duke of Wellington, Sir Astley Cooper and Sir Henry Halford. He immediately commenced to syringe the ear. During the operation Mr. Peel became rather too inquisitive as to the nature of his complaint, its situation, and the *modus operandi* of the remedy. Curtis was in a very difficult position, but his natural shrewdness and imperturbable coolness made him equal to the occasion. "I saw," he said, "that I must stop this inconvenient questioning, so putting the point of the syringe by the side of the passage I gave him a dig and said, 'Mr. Peel, if you don't hold your tongue I shall certainly do you a mischief.' He was as dumb as an oyster afterwards."

### SIMPLE OPENING OF THE MASTOID ANTRUM.

The first suggestion that the mastoid process should be artificially opened appears to have been made by Johannes Riolanus (1649). One of the passages in his writings which refers to the procedure is very curious. He describes the mastoid cells as being like the cells of a honeycomb, and then continues :—"Vesalius compared it to a large cave; on account of its being full of air, it happens when the air, which should always be still, is stirred up by the impact of a fresh air current, that the ears buzz continually. But how can we give exit to the air which is such a source of trouble? There is certainly no other way of doing this except trephining the mastoid process."

The opening suggested was to be a kind of air vent or noise vent for the relief of tinnitus aurium in cases of Eustachian obstruction, and this writer and others who followed, including Rolfinck, though they suggested making the opening, yet never themselves performed the operation.

Valsalva, 1740, and Heuermann, 1757, were the first surgeons to record the use of spontaneous openings in the mastoid process for the purpose of treating otorrhœa.

Each of them reported a case in which he syringed through such openings into the tympanum.

The first surgeon, however, to perform and definitely advocate opening the mastoid process for the evacuation of pus was the celebrated Jean Louis Petit (b. 1674, d. 1760). He recommended that abscesses in the mastoid diploë should be opened " from the very first day of their formation," and he taught that we should not " wait until they have destroyed the bone, for the patient is always in danger" (Ballance). He used a so-called " trepan exfoliatif," which bored a hole but did not remove a disc of bone. It is to be regretted that this most sound advice was not accepted by the profession for a century. He preached a gospel of reform which surgeons had not the sense to accept.

About sixteen years later a Prussian regimental surgeon, named Jasser, in 1776, unacquainted with Petit's writing, operated successfully on the mastoid of a soldier. The story of Jasser's case may be epitomised from the account given by Sir C. Ballance as follows :—

The original account is in a German book published in 1782.

A recruit came to the regiment of which Jasser was the surgeon, with hearing lost on the left side and bad on the right, and with offensive discharge from both ears. The colonel was present when the recruit was examined. He raised his stick saying that the stick was the proper instrument with which to restore the hearing and drive away the discharge, so to duty the recruit had to go. Several times in the next few weeks the man came to hospital with pain in the right ear. He was bled, purged, blistered and leeched without getting relief. At last an incision was made over the right mastoid. A little thin pus escaped. Later, no relief having been obtained, the mastoid wound was enlarged, a bare spot of bone felt, and on probing the probe entered the mastoid through a small hole. Jasser was rather alarmed and injected a medicinal concoction, the only thing handy. The nozzle of the syringe fitted the hole tightly. When the injection was made the patient called out "Good God, something is running through my ear into my head." Some of the fluid came out of the right nostril. Jasser was startled. He repeated the injection. Much pus came from the meatus. Jasser asked the man how he felt. He replied, "God be thanked for ever, my earache is going!" He then slept for ten hours. The wound healed in three weeks with improved hearing.

The "Jasser Operation" was widely discussed, but apparently was not repeated for ten years, when it was performed by a surgeon named Fielitz. Whiting writes somewhat scathingly of Fielitz, as follows:—"He was possessed of the requisite fortitude to insure not only the performance of the operation, but, to judge by his subsequent report, endowed as well with the needful assurance to warrant the unequivocal success of any step which he might attempt, providing the publishing of the same were entrusted to him." He operated on five mastoids of three very deaf patients, only one of them having otorrhœa. All are reported to have recovered quickly with complete restoration of hearing. This result is incredible.

The true principles upon which the mastoid operation should be performed had been lost sight of, and instead the operation was resorted to as a cure for deafness. One or two more surgeons tried it with indifferent or bad results, including Kolpin, who operated on the celebrated Danish Court Physician, Johan Just von Berger, for deafness and tinnitus in 1791. The patient died, and was regarded by many as having died "a martyr to the operation of perforation of the mastoid." This misfortune appears to have checked the progress of mastoid surgery for years. As a matter of fact an autopsy on von Berger seemed to show that he died from meningitis secondary to the scalp and skull wound, and that the dura had not been injured by the operation.

So the Jasser operation after a series of failures died out.

Its practice had come to be based on false ideas, and mastoid surgery suffered eclipse. Its consideration, however, had served as a stimulus to anatomical and physiological research.

In 1838 J. E. Dezeimeris collected nine cases in which the operation of opening the mastoid had been performed, some of which have been mentioned above, and he defended the operation.

One turns in England with special interest to the opinions held by Wilde and Toynbee on this subject. Wilde, in his work on *Aural Surgery*, published in 1853, wrote:—"As the success attending this procedure must be very doubtful, and the hazard very great, it is never resorted to in the present day." He recommends making the famous incision associated with his name (the Wilde incision), "should the mastoid process, or the parts covering it, become engaged, and the methods already recommended fail to give relief."

Toynbee thought it would be right to perforate the mastoid in cases where pent-up matter was causing "such urgent and serious symptoms as are likely, if not relieved, to terminate in death." He had never performed the operation, but would not scruple to do so in the circumstances mentioned. In 1860 a French surgeon, Forget, advocated the operation. Then von Tröltsch, in 1861, showed that he appreciated the true surgical principles on which the mastoid should be opened. He pointed out the various dangers which arise from delay in evacuating pus from the bone, and during the next few years cases were from time to time operated on. In 1868 Jacoby described the indications for the operation, and included among them obstinate otorrhœa without signs of mastoid implication. His method, and that of all other surgeons, however, was merely to make a hole with a drill.

### THE SCHWARTZE OPERATION.

Now, at long last, we come to Schwartze. According to Buck there had been only thirty-five such operations recorded up to 1873, when Schwartze began to publish his work on the subject. Sir C. Ballance writes :—" It is to the scientific insight and work of Schwartze and his assistant, A. Eysell, that we are mainly indebted for the first systematic account of the operation as a scientific procedure to be performed on a definite plan and under definite conditions."

Schwartze laid stress on irrigating through the mastoid opening and antrum, into the tympanum and out through the meatus, a step we do not now lay stress upon. We can say, with full justification, that the ordinary mastoid operation of to-day, as performed in acute cases and as a first step in more complete operations, is that of Schwartze. The operation as usually done is rather more extensive than that originally described, and has been modified slightly in various ways, but it remains essentially Schwartze's. One must admit, however, in fairness, that in all probability Jacoby and von Tröltsch, both of whom were working at the subject about the same time, had a share " in defining the plan of operation which came to be known as Schwartze's " (Ballance).

### THE RADICAL MASTOID OPERATION.

The next advances made in the surgery of this region, were those which have led to the operation now known as the Radical Mastoid Operation.

It was soon found that the operation of Schwartze, successful though it was in the treatment of acute mastoid inflammation, was not usually successful when tried for the cure of obstinate chronic middle-ear suppuration with more or less foul discharge. This was due to the fact that in those cases there existed with the mastoid disease caries of other parts, such as the bony walls of the tympanum, the ossicles and the osseous meatus. It was the attempt to treat these obstinate cases effectively which led gradually to the evolution of the radical operation, in which not only the mastoid antrum and cells are freely laid open, but also the whole tympanic cavity is opened up and cleared of its contents.

It was the general surgeon Küster who gave the first impetus to this extension of the operation in 1889, when he recommended that the removal of the posterior wall of the osseous meatus should be added to the procedures of Schwartze. That was the first step in the evolution of the radical operation. In the same year Von Bergmann extended the operation still further by removing the outer attic wall. Küster's principle was, in his own words, as quoted by Ballance, "to open up the bone freely so that it can be readily scrutinised, to clear away all disease and thus so fully to expose the source of suppuration that the pus is nowhere checked in its outflow." Next came Stacke and Zaufal, who planned systematic methods of laying open into one easily accessible cavity, for drainage and dressing, of the antrum, attic and tympanum. Zaufal worked from behind forwards; Stacke opened the attic first and then worked backwards into the antrum. The Zaufal method is that most commonly used. I have myself never used the Stacke mode of approach. Stacke was the first to introduce the plastic procedure in which the posterior wall of the membranous meatus is divided to form one or more flaps, and the flap or flaps displaced backwards into the bony cavities. This prevents cicatricial narrowing of the meatus, and gives a starting point for the spread of epidermis over the raw surfaces of the bony cavity which has been made. Many modifications of this plastic part of the operation have been employed.

When I first began to perform radical mastoid operations I tried the technique described by Sir Charles Ballance in 1900. The mastoid incision was re-opened some time after the first operation, under an anæsthetic, Thiersch's skin grafts were applied to cover the walls of the bony cavity and the grafts covered with gold leaf as a protective. I showed cases at this Society in which these methods had yielded gratifying results. But I soon came to use simpler methods of performing the plastic part of the operation and to omit skin grafting. I have still an open mind on the question of applying skin grafts, but I do not like to subject my patients to two operations if as good an end result can be obtained with one by the exercise of a little patience. Soon after I had taken charge of the ear department at the Bristol General Hospital I paid a holiday visit to Politzer's Clinic in Vienna. A rather curious incident was that one day Mr. C. A. Ballance appeared and operated on a patient by his method, with Politzer and his assistants looking on as interested spectators. I must admit I could not get a good view owing to the number of students present. One student standing near me, a foreigner, seemed much impressed with the coolness and imperturbability of the operator, which he considered was characteristically English. The post-graduate instruction in the clinic was in my opinion excellent. For instance, a class was formed for practising aural operations of the cadaver. Each student performed mastoid operations and could later obtain the temporal bone of the head on which he had operated. I brought one home with me, but I feel sure it was not the one I had operated on myself. For one reason the operation had been crudely performed, more crudely than I believed I had done it. The supply of cadavers for these operative classes seemed limitless at the Allgemeines Krankenhaus.

I have no recollection of ever seeing a mastoid operation done at University College Hospital from November, 1886, to November, 1889. In 1890, when I was House Surgeon at the Royal Berkshire Hospital, Reading, I saw Wilde's incision made for mastoiditis. To-day we should all agree with Whiting's statement that "As a general proposition we may affirm that whenever Wilde's incision is indicated a mastoid operation is imperative." Also we should agree with the same writer's remark that "Probably no surgical measure which had its foundation upon such frail anatomical and surgical supports ever enjoyed the distinction of a vogue so popular and prolonged as 'Wilde's Incision.'"

### THE OPERATION FOR SINUS THROMBOSIS.

The last extension of temporal bone surgery I wish to notice is that for dealing with infective lateral sinus thrombosis.

It is rather curious to find that infective sinus thrombosis of otitic origin was not recognised until long after otitic brain abscess and meningitis. The first observations on the subject are those of Hooper in 1826, and of Abercrombie in 1835. In 1840 Bruce read a paper in Liverpool on the subject.

We turn again with interest to Wilde's book on *Aural Surgery*, published in 1853. He wrote as follows :—"These cerebral symptoms do not, however, in every case appear to be the immediate cause of death. I remember two cases in particular and I suppose they are the types of many others of the same class, where the lungs became affected in the later stage of the disease, and in which the thoracic affection seemed to be the immediate cause of dissolution . . . In such cases I am inclined to think that the disease of the parts of the bone extends to the lateral sinus and induces purulent infection of the venous system, which eventuates in disease of the lung."

Lebert in 1856 gave the first accurate description of this complication based on his own and older observations. He considered the prognosis absolutely unfavourable.

With reference to the treatment of the condition Sir C. Ballance writes as follows :—" The principle on which it is based was first successfully applied in the treatment of venous infections arising in other parts of the body. Kraussold, for example, in 1878, in a case of suppurating thrombosis of the femoral vein, following amputation through the thigh, opened the ligatured end of the vein, cleared out the clot, tied the femoral vein at the level of Poupart's ligament and excised 2 to 3 cm. of the intervening portion of the vein. The patient recovered. . . . He suggested that a similar operation might be required for the internal jugular vein, or any large vein, for the cure of sepsis following operations. He gives Lee the credit of having been the first to tie a vein to arrest septic infection. Lee, in a case of septic infection spreading from the hand, divided the cephalic vein subcutaneously and secured the ends by acupressure."

But to Zaúfal belongs the credit of having first suggested that lateral sinus thrombosis of otitic origin should be treated by ligature of the jugular vein and by opening the sinus. Zaufal's words as rendered in Sir C. Ballance's work, are the following :—" It is not the thrombus, but the breaking down of the thrombus which causes fatality. Unfortunately, we cannot diagnose infective sinus thrombosis until after rigors and other signs, which indicate that infective particles have been carried into the blood stream, have appeared. Can we get rid of such a collection ? The case I have brought forward, in which the vena jugularis on the cardiac side was occluded by uninfected clot, is like a finger-post pointing the way. In such cases I think not only the mastoid operation but ligature of the jugular vein and sometimes opening up part of the sinus itself would be useful." This was written in 1880.

In 1884 Zaufal recommended that bone should be chiselled away and the sinus opened so that infected material within it might be cleared out by antiseptic injections. This can only be done safely if the jugular vein is blocked at the foramen jugulare or lower down, so that fluid injected cannot reach the innominate vein and the right side of the heart. If this is not the case, the jugular vein must be tied below the diseased part, and " so the highway for infective particles into the blood stream occluded. If the jugular vein above the ligature contains infective clot it must be opened also and irrigated."

Voss, writing in 1910, remarks that German aural surgeons spent nearly ten years in academic discussion of Zaufal's recommendations without practising them, and then an English surgeon, unacquainted with those recommendations, was the first to convert similar ideas into action. The surgeon was Sir Arbuthnot Lane, whose first operation of the kind was performed in 1888, and the case described at the Clinical Society in April, 1889. In May, 1889, Sir C. Ballance, unaware of the work of Zaufal or of Lane, performed a similar operation, and published his first paper on the subject in March, 1890. Eleven years later, in 1901, Viereck published the results of 170 such operations, of which 62 were considered to have been too late to have any chance of success. Of the remaining 108 cases 89 were successful. Ballance was the first to advise dividing the vein between two ligatures, " so that the upper septic portion of the jugular may be completely isolated from the lower, which remains an integral portion of the vascular mechanism."

When Sir Arbuthnot Lane ligatured the jugular vein for the first time in history for the treatment of lateral sinus thrombosis, he is said to have been influenced to do so by a suggestion of Sir Victor Horsley's. It is interesting, therefore, to consider Horsley's contribution to the subject.

In 1886, two years before Lane operated, Horsley read a paper at a meeting of the Clinical Society of London on a "Case of Suppuration in the Mastoid Cells," and in that paper suggested dividing the jugular vein to prevent septic emboli passing to the thoracic viscera. Horsley's case was interesting, too, as a record of recovery after such an embolism had occurred.

I propose, therefore, to read to you an epitome of Horsley's records of the case, and to give you verbatim some of the remarks made on the subject of ligaturing the jugular vein.

The patient was a young woman of seventeen years of age, admitted to University College Hospital after being ill for a fortnight. She had profuse discharge from her right ear, and swelling and redness over the mastoid process. She had two operations on the mastoid, the bone being freely laid open and much foul pus evacuated. Ten days after admission, and a few days after the second operation, she was seized with sudden severe precordial pain and great dyspnœa. A temporary bruit was heard at the apex of the heart. In twenty-five minutes the pain shifted to a position beneath the left shoulder-blade. Next day there was a patch of dullness and tubular breathing opposite the lower angle of the left scapula. The patient recovered.

The lateral sinus, I must point out, was not treated, but was assumed by Horsley to have been the source of the pulmonary embolus.

Horsley comments as follows on the case :—

"There also, of course, comes in the question how far we can limit the extent of the thrombosis of the sinus if it has once begun. From consideration of the dates given in the foregoing report it will, I think, be conceded that the serious state of the patient (stupor, temperature 106°, etc.), which led me to so freely open up the mastoid and tympanic cavities, was caused by such thrombosis having commenced. This being so, it is clear that the thorough disinfection and drainage of the inflamed bone checked the osteitis and consequently the progress of the thrombosis caused thereby.

"Supposing this to be always possible, there still remains the problem how to prevent embolism of the thoracic viscera. . . . The solution of this problem is a simple matter enough, looking at it from the merely mechanical point of view, resolving itself, of course, into the really not very serious operation of ligature of the jugular vein in the middle of the neck. . . . At the first blush it will seem, perhaps, to be a very crude proposal, but a little consideration of the facts which led me to suggest it will, I think, alter this adverse criticism."

Horsley then discusses the question of the spread of the thrombosis backwards towards the torcular, and concludes that this would not be more likely to occur after ligature than before. It is noticeable that the idea of opening up the lateral sinus and clearing out the septic clot did not enter Horsley's mind, and in this and other respects his suggestions for the treatment of sinus thrombosis were less complete than the earlier suggestions of Zaufal recorded above. [1]

Now, gentlemen, my excursion into the field of Surgical History is finished. You will have noted that the discoveries and inventions to which I have alluded were not made by men of transcendent genius, not by men of the calibre of Leonardo da Vinci, Newton, Pasteur or Lister, but at least

[1] A series of lantern illustrations of the mastoid operation and its evolution were next shown and the following portraits: Jean Louis Petit (b. 1674, d. 1750); E. Siegle (b. 1833, d. 1900); Heinrich Adolf Rinne (b. 1819, d. 1868); Joseph Toynbee (b. 1815, d. 1866); Sir William Wilde (b. 1815, d. 1876); Hermann Schwartze (b. 1837, d. 1910); Emil Zaufal (b. 1837, d. 1910); Ménière d'Angers (b. 1799, d. 1862); Sir Victor Horsley (b. 1857, d. 1916).

they were made by men who worked well and made lasting contributions to our science and art. By men, therefore, whose deeds are worthy of our admiration and emulation. Many of us may not be so well equipped by nature as they were to make notable contributions to science, but each one of us can do something. As an encouragement to effort in this direction, let me read you two brief extracts from the writings of men great in literary achievement. First an exhortation taken from Carlyle's *Sartor Resartus*, where it is written :—

"Produce! Produce! Were it but the pitifullest infinitesimal fraction of a Product, produce it, in God's name! 'Tis the utmost thou hast in thee; out with it, then. Up, up! Whatsoever thy hand findeth to do, do it with thy whole might. Work while it is called to-day."

My second quotation is from Kipling's poem, "The Glory of the Garden." The poem begins, you will remember, with the words, "Our England is a Garden," but let us consider it as though the opening words were, Our Science is a garden, a garden which needs constant work to maintain its glory. I must only quote two verses, but the whole poem would well bear the application I am suggesting :—

"There 's not a pair of legs so thin, there 's not a head so thick,
There 's not a hand so weak and white, nor yet a heart so sick,
But it can find some needful job that 's crying to be done,
For the Glory of the Garden glorifieth everyone.

"Then seek your job with thankfulness and work till further orders,
If it 's only netting strawberries or killing slugs on borders;
And when your back stops aching and your hands begin to harden,
You will find yourself a partner in the Glory of the Garden."

Lastly, may I appeal to you, members of this Society, to cultivate during the ensuing session, with special sympathy and special zeal, one flower-bed in that Garden of Science, that of the Bristol Medico-Chirurgical Society.

